# Skeletal Muscle Mass Indices in Healthy Young Mexican Adults Aged 20–40 Years: Implications for Diagnoses of Sarcopenia in the Elderly Population

**DOI:** 10.1155/2014/672158

**Published:** 2014-02-06

**Authors:** H. Alemán-Mateo, Roxana E. Ruiz Valenzuela

**Affiliations:** Coordinación de Nutrición, Centro de Investigación en Alimentación y Desarrollo (CIAD), A.C. Carretera a La Victoria Km. 0.6, 83304 Hermosillo, Sonora, Mexico

## Abstract

*Background and Objectives*. Skeletal muscle and skeletal muscle indices in young adults from developing countries are sparse. Indices and the corresponding cut-off points can be a reference for diagnoses of sarcopenia. This study assessed skeletal muscle using dual-energy X-ray absorptiometry (DXA) in healthy male and female subjects aged 20–40 years and compared their appendicular skeletal muscle mass (ASM) and total-body skeletal muscle (TBSM) indices using certain cut-off points published in the literature. *Methods*. A sample of 216 healthy adults men and women from northwest Mexico was included. Body composition was assessed by DXA and several published DXA-derived skeletal muscle indices were compared. *Results*. Both, ASM and TBSM were higher in men compared to the women group (23.0 ± 3.4 versus 15.9 ± 1.6 kg; *P* < 0.05 and 26.5 ± 4.1 versus 16.9 ± 1.9 kg; *P* < 0.05, resp.). These differences were also valid for both indices. When derived cut-off points were compared with the most reported indices, significant differences were found. *Interpretation and Conclusion*. Published cut-off points from Caucasians are higher than cut-off point derived in this sample of Mexican subjects. The new DXA-derived cut-off points for ASM proposed herein may improve diagnoses of sarcopenia in the geriatric Mexican population.

## 1. Introduction

Skeletal muscle (SM) plays a central role in many biological functions, such as movement and metabolism, so disruptions in this component of body composition can have a marketed influence on health and disease [1]. Also, we cannot ignore the influence of this factor on nutritional status and functionality at older ages. Skeletal muscle in both men and women subjects remains relatively constant during the third and fourth decades of life but begins to decline noticeably at ~45 years of age [2–4]. It has been suggested that the maintaining muscle mass in old age is a key factor for conserving physical capacity and enabling independent lifestyles in old age. Human skeletal muscle mass depends basically on stature, weight, age, and gender. In a published study these factors explained >80% between-individual within-ethnic group (African-Americans and Caucasians) differences in SM mass [3].

The loss of SM is a common problem in older adults from around the world that reaches a prevalence ranging from 7% to over 50% [5]. At one time, the age-related loss of skeletal muscle was called sarcopenia, but now its definition is not limited only to the loss of muscle mass. Sarcopenia comprised the loss both muscle mass and strength with a risk of adverse outcomes such as physical disability, poor quality of life, and even death [5]. In terms of diagnoses, sarcopenia is characterized by low muscle mass plus low muscle strength or low physical performance. To establish “low muscle mass”, some researchers have assessed the total or appendicular skeletal muscle in young adults (the age range in which muscle reaches a plateau and then remains relatively constant). It is important to highlight that the absolute skeletal muscle mass (kg) is normally converted to skeletal muscle indices. Baumgartner et al. [6] summed the muscle mass of the four limbs assessed by DXA and used the term appendicular skeletal muscle mass (ASM) to refer to this sum. They then defined the appendicular skeletal muscle mass index (ASMI) as ASM/height^2^ (kg/m^2^). ASMI two-standard deviations below the mean ASMI of healthy young male and female reference groups were defined as the gender-specific cut-off points for sarcopenia (original term used).

Several cut-off points based on the ASMI or total-body SM mass index (TBSMI) have been derived [6–13]; however, these cut-off points depend on several factors: the measurement technique used, age range, sex, ethnicity, and the availability suitable references studies. For the Mexican case, unfortunately, no such reference SM data exist, so the few published studies on sarcopenia have used a cut-off point based on an ASMI derived from a young Caucasian population [14]; even though could well bias the prevalence of the loss of skeletal muscle or sarcopenia. In addition, several studies have shown significant differences in the prevalence of sarcopenia when different indices are used interchangeably or are applied to populations distinct from the original sex-specific group of subjects [15]. Due to these issues, the recommendation is that these indices would ideally refer to sex-specific ethnic group populations [5]. Therefore, this study assessed skeletal muscle using dual-energy X-ray absorptiometry in healthy male and female subjects aged 20–40 years and compared their ASM and TBSM indices using certain cut-off points published in the literature.

## 2. Materials and Methods

### 2.1. Study Design and Subjects

This is a cross-sectional, nonprobability study that included 216 apparently healthy adult men and women from northwest Mexico. All volunteers were selected on the bases of inclusion and exclusion criteria. All men and (nonpregnant) female subjects were between 20 and 40 years of age, were apparently healthy by self-report, and were either born in the state of Sonora, Mexico or had lived there for at least five years. Subjects were excluded if their body weight or height was outside the DXA bed dimensions. Subjects who reported changes in body weight in the last six months due primary to caloric restriction or increased physical activity were not included. For safety reason, pregnant women were not invited to participate and those who were positive on a pregnancy test (DIAGMEX commercial test) were not included for the DXA measurements. Also, volunteers who were taking medications that affect body composition and those with protein supplementation were excluded. Most volunteers had lifestyles that involved only light occupational and leisure time activities. Finally, trained technicians took all anthropometric and body composition measurements of subjects under fasting conditions or at least two hours after subject's most recent meal or drink.

### 2.2. Anthropometry

#### 2.2.1. Body Weight

This variable was measured with subjects dressed in light clothing and shoes less (avoiding extra weight such as wallets, money, and phones). The Digital Electronic Scale (PV-150 K DNA, Japan) used was calibrated before taking measurements.

#### 2.2.2. Height

This was measured without shoes and according to our standardized protocol, using a calibrated Holtain stadiometer (Holtain Ltd, Dyfed, UK).

#### 2.2.3. Body Mass Index

BMI was calculated on the basis of body weight and height measurements and classified according to WHO's 1997 cut-off points [16].

### 2.3. Body Composition

The evaluation of body composition including the lean tissue was carried out under fasting conditions or at least two hours after the most recent light meal or drinks, following established guidelines and using a DXA (DPX-MD+; GE Lunar Madison, WI, USA). Regional body composition components were determined from the DXA scans, following the recommended anatomical landmarks. The sum of nonfat plus nonbone tissue in both arms and legs was used to represent ASM [17]. Limb skeletal muscle mass represents 75% of total body skeletal muscle [18]. Other body composition components, such as total body lean tissue and fat mass, were also assessed. From DXA-derived ASM, total-body skeletal muscle mass was predicted using Kim's equation [19].

### 2.4. Skeletal Muscle Mass Indices and Cut-Off Points

Two indices were calculated: ASMI as ASM/height^2^ (kg/m^2^) and TBSMI as TBSM/height^2^ (kg/m^2^). Two cut-off points were established considering two standard deviations below the mean of both indices derived from the SM assessed in a sample of healthy young man and women from northwest Mexico.

### 2.5. Skeletal Muscle Mass Indices and Cut-Off Points Derived from the Other Populations

For this study, Baumgartner et al., Sanada et al., Lau et al., Wen et al., and Kim et al.'s sex-specific cut-off points for ASMI [6–10] and Lau et al., Tichet et al., Chien et al., and Masanes et al. [8, 11–13] sex-specific cut-off points for TBSMI were used for purposes of comparison.

### 2.6. Statistical Analyses

Results are presented as mean standard deviations by sex, while significant differences between man and women were tested by a two-sample *t*-test. A one-sample *t*-test was also used to test the significant differences between our sex-specific cut-off points compared to the different published ASMI and TBSMI cut-off points derived from a young adult ethnic group populations.

## 3. Results

This study included 216 subjects, 63% were men and the rest women. Average was 27.6 ± 5.2 years, with a range of 18 to 40 years. The mean BMI was 24.7 kg/m^2^ which falls into the “normal” range according to the WHO's classification. Regarding the anthropometric data collected, a significant gender effect was found. As expected, the male group weighed 18 kg more than the female group (*P* < 0.001), and height in former was higher than in the latter by an average 13 cm (*P* < 0.001). These differences were reflected significantly in the BMI measurements, and similar results were found for the body composition components assessed. Both, ASM and TBSM were higher in men compared to the mean value for the women's group. These differences were also valid for both indices. However, total and truncal fat were significantly higher in the women group (Table 1).

Table 2 shows the effect of gender on the new proposed cut-off points based on two standard deviations below the mean value of both indices, derived from the sample included. Both indices were significantly higher in the men's group. When these cut-off points were compared to the most often reported indices in the literature, significant differences were found for all indices derived from other sex-specific ethnic groups, except those from a sample of Chinese and Hong Kong men and a sample of Chinese women (Table 3).

## 4. Discussion

This is the first study to use DXA with a sample of young Latin American adults to determine indices based on our skeletal muscle estimates. The cut-off points derived from this sample of male and female Mexican subjects may improve diagnoses of sarcopenia in the Mexican geriatric population or among Latin American people with similar characteristics to those in the sample assessed herein. Overall results confirm the effect of gender on most of the anthropometric and body composition components and DXA-derived indices. In addition, our results showed significant differences with most of the sex-specific population indices published. These results emphasize that these published indices cannot be used interchangeably.

The expected results on the differences between the derived indices from this sample and those published previously can be related to the determinants of differences in body composition, especially skeletal muscle. It is well recognized that skeletal muscle depends on age, gender, ethnicity, body weight, height, and longer extremities [3, 20, 21]; therefore skeletal muscle indices could depend on the same factors. In order to assess the clinical impact of those indices physical performance test should be assess in order to look the association between the sex-specific cut-off points and physical disabilities in different ethnic groups, but we cannot ignore the effect of the techniques used to measure muscle mass since its highly likely that all these determinants may influence the indices.

The indices derived from some Caucasian populations are higher (i.e., Baumgarnert et al.'s cut-off points of 7.26 and 5.45 for young man and women adults, resp., [6]) because, in general, Caucasian people are taller than Latina American or Asian populations, while in the case of the African-American people we find significantly greater skeletal muscle mass [20] and it has been reported longer appendicular bone lengths [22, 23] compared to Caucasian subjects. If we compared the African-Americans with other ethnic groups, the results would probably not change. For all these reasons, plus the high prevalence of loss of skeletal muscle and sarcopenia around the world, and the clinical impact of these conditions, more research is needed to obtain good reference studies that will establish valid SM cut-off points for populations worldwide and to translate this information to the clinical practice in order to detect patients with low muscle mass or sarcopenia.

The clinical implication of the results of this study is that the interchangeably use of these cut-off points may under- or overestimate real prevalence of loss of skeletal muscle mass or sarcopenia in older people in Mexico or other Latin American countries. Specifically, if we were to use the cut-off points reported by Baumgartner et al., a significant underestimation of the prevalence of the loss of skeletal muscle mass or sarcopenia would be reported for older people, characterized by low skeletal muscle mass or ASMI [20]. Similarly, after applying the ASMI criteria for sarcopenia in a study of an elderly Chinese population Wen et al. [9] concluded that this method may not be appropriate for diagnosing sarcopenia in that group because no older adults in the study were diagnosed with this condition. The criteria based on the skeletal muscle index proposed by Janssen et al. [24] showed greater discriminating power in identifying persons with low handgrip strength. Considering the findings from Wen et al. [9] in addition to having sex-specific cut-off points for different populations or ethnic groups it is clear that the use of other criteria for sarcopenia and clinical assessments of functionality is required for target population.

With respect to clinical implications of the results of this study mentioned before, it is important to consider that skeletal muscle is a required component for diagnoses of sarcopenia [5]. Therefore, the cut-off points for muscle strength in the young reference population or low physical performance in elderly participant in addition to the sex-specific cut-off points for skeletal muscle may improve diagnosis of sarcopenia in the target elderly population.

### 4.1. Strengths and Limitations

The strength of this study rests on the use of DXA to assess lean tissue, since this technique accurately measures ASM. The comparison with other cut-off points based on DXA-derived ASM is another strength, although the use of a nonrandom sampling method and the nonpopulation-based nature of this work limit the ability to generalize results. For these reasons, future population-based studies are needed to obtain better reference cut-off points for ASM and other body composition components in the young Mexican adult population. Also, we are aware that the reported cut-off points based on ASM or TBSM derived from a young adults population adjusted to the dimensions of the DXA bed could be another important limitation, as individuals taller than 1.96 meters could not be assessed by DXA. The estimates of TBSMI using a published DXA-equation derived from a Caucasian population and the absence of a gold standard method to assess TBSM could be an additional limitation.

## 5. Conclusion

In conclusion, this study confirms an effect of sex on several body composition components and DXA-derived indices such as ASMI and TBSMI in the sample of young adult assessed. Published cut-off points from Caucasians are higher than those derived in this sample of men and women Mexican subjects so the use of those published indices interchangeably could underestimate the prevalence of the loss of skeletal muscle mass or sarcopenia in older people characterized by low skeletal muscle mass or ASMI. The new DXA-derived cut-off points for ASM proposed herein may improve diagnoses of sarcopenia in the geriatric Mexican population or that of other Latin American countries with similar characteristics to those assessed in our study sample.

## Figures and Tables

**Table 1:** Anthropometry and body composition components of healthy young Mexican adults.

Variables	Men (*n* = 136)	Women (*n* = 80)	Both men and women
Age, (years)	27.3 ± 5.0	28.2 ± 5.6	27.6 ± 5.2
Weight, (kg)	77.9 ± 13.0	60.4 ± 8.5*	71.5 ± 14.3
Height, (m)	1.74 ± 0.1	1.61 ± 0.04*	1.69 ± 0.08
BMI, (kg/m^2^)	25.7 ± 3.6	23.2 ± 3.1*	24.7 ± 3.6
ASM, (kg)	23.0 ± 3.4	15.9 ± 1.6*	20.1 ± 4.7
ASMI, (kg/m^2^)	7.5 ± 0.8	5.8 ± 0.5*	6.6 ± 1.1
TBSM, (kg)	26.5 ± 4.1	16.9 ± 1.9*	22.9 ± 5.7
TBSMI, (kg/m^2^)	8.6 ± 1.0	6.5 ± 0.6*	7.8 ± 1.3
FFM, (kg)	52.9 ± 7.1	34.2 ± 3.2*	46.0 ± 10.8
Fat mass, (kg)	20.5 ± 8.5	22.2 ± 6.6**	21.2 ± 4.9
Truncal fat, (kg)	13.1 ± 5.4	11.3 ± 3.8**	12.4 ± 7.9

BMI: body mass index; ASM: appendicular skeletal muscle mass; ASMI: appendicular skeletal muscle mass index; TBSM: total-body skeletal muscle mass; TBSMI: total-body skeletal muscle mass index; FFM: fat-free mass (total lean tissue plus total bone mineral content); *P < 0.001; **P < 0.05.

**Table 2:** Sex-effect on the proposed cut-off points based on two standard deviations below the mean value of the ASMI and TBSMI derived from healthy young Mexican adults.

Variables	Men (*n* = 136)	Women (*n* = 80)	*P* value
ASMI, (kg/m^2^)	5.86	4.72	0.0001
TBSMI, (kg/m^2^)	6.63	5.22	0.0001

ASMI: appendicular skeletal muscle mass index; TBSMI: total-body skeletal muscle mass index.

**Table 3:** Comparison between the proposed sex-specific cut-off points against some published indices from around the world.

Indices by authors	Sex-specific cut-off points	
	Men	Women
ASMI, (kg/m^2^)		
Alemán and Ruiz, 2014	5.86	4.72
Baumgartner et al., 1988	7.26*	5.45*
Sanada et al., 2010 [7]	6.87*	5.46*
Lau et al., 2005 [8]	5.72*	4.82
Wen et al., 2011 [9]	5.80	4.30*
Kim et al., 2009 [10]	7.40*	5.10*
TBSMI, (kg/m^2^)		
Alemán and Ruiz, 2014	6.71	5.22
Lau et al., 2005 [8]	7.66*	6.40*
Tichet et al., 2008 [11]	8.60*	6.20*
Chien et al., 2008 [12]	8.90*	6.50*
Mesanes et al., 2012	8.31*	6.68*

ASMI: appendicular skeletal muscle mass index; TBSMI: total-body skeletal muscle mass index.

*P < 0.001.
